# Uncovering a novel *SERPING1* pathogenic variant: insights into the aggregation of C1-INH in hereditary angioedema

**DOI:** 10.1186/s13023-024-03306-7

**Published:** 2024-09-13

**Authors:** Lingxi Jiang, Chao Dai, Suyang Duan, Tingting Wang, Chunbao Xie, Luhan Zhang, Zimeng Ye, Xiumei Ma, Yi Shi

**Affiliations:** 1 Sichuan Provincial Key Laboratory for Human Disease Gene Study and the Center for Medical Genetics, Department of Laboratory Medicine, Sichuan Academy of Medical Sciences & Sichuan Provincial People’s Hospital, University of Electronic Science and Technology of China, 32 The First Ring Road West 2, Chengdu, Sichuan 610072 China; 2 Department of Ophthalmology, Sichuan Provincial People’s Hospital, University of Electronic Science and Technology of China, Chengdu, 610072 China; 3 Research Unit for Blindness Prevention of Chinese Academy of Medical Sciences (2019RU026), Sichuan Academy of Medical Sciences and Sichuan Provincial People’s Hospital, Chengdu, 610072 China; 4 School of Medicine, University of Sydney, Camperdown, NSW 2050, 2006 Australia; 5 Health Management center, Sichuan Provincial people’s Hospital, Chengdu, 610072 China

**Keywords:** Apoptosis, Ca^2+^ overload, C1-INH, Hereditary angioedema, *SERPING1* pathogenic variant

## Abstract

**Background:**

Hereditary angioedema (HAE) is a rare autosomal dominant genetic disease characterized by recurrent edema and a potentially fatal risk. Despite its severity, there is a notable lack of effective methods for predicting and preventing HAE attacks. This study aims to thoroughly investigate the underlying pathological mechanisms of HAE and identify potential biomarkers that could aid in its prediction and prevention.

**Results:**

In our investigation, we have discovered a novel pathogenic variant of the *SERPING1* gene, specifically c.708T > G, in a Han family affected by HAE. Our observations indicate that this variant leads to an increase in the accumulation of C1-INH within the endoplasmic reticulum (ER), resulting in the upregulation of GRP75 protein expression. This cascade of events resulted in Ca^2+^ overload, disruption of mitochondrial structure and function, and eventually triggered apoptosis. Using siRNA to knock down GRP75 mitigates cellular calcium overload and mitochondrial damage induced by the *SERPING1* mutation.

**Conclusion:**

Based on our findings, we propose that the detection of intracellular Ca^2+^ concentration could serve as a valuable biomarker for predicting acute attacks of HAE in patients. This discovery holds significant implications for the development of more targeted and effective strategies in the management of HAE.

## Introduction

Hereditary angioedema (HAE) is a rare and life-threatening disorder distinguished by recurrent episodes of angioedema that primarily affect subcutaneous or mucosal tissues. HAE exhibits spontaneous resolution with a period of 3–5 days and displays inherent self-recovery properties [1]. The occurrence of edema in critical regions of HAE always manifests in the face, throat, genital, or peripheral abdomen, posing significant risks and potentially leading to fatalities in up to 28% of affected individuals [2]. Various pathogenic genes associated with HAE have been reported, including Serine Protease Inhibitor G1 (*SERPING1*), Factor XII (*F12*), Plasminogen (*PLG*), myoferlin gene mutation (*MYOF*), kininogen-1 (*KNG1*), heparan sulfate-glucosamine 3-O-sulfotransferase 6 (*HS3ST6*), and Angiopoietin 1 (*ANGPT 1*) [3]. Notely, *SERPING1* has garnered significant attention, as approximately 85% of HAE patients exhibit *SERPING1* gene mutations [4, 5].

As a member of the serine protease inhibitor superfamily, CI-INH, encoded by *SERPING1*, is the only complement inhibitor and has a direct effect on the occurrence of HAE [6, 7]. Clinical observations indicate that approximately 40% of the normal secretion of C1-INH is sufficient to maintain the stability of the complement system [8]. However, when C1-INH secretion falls below 30% [9–11], complement disorder occurs. Recent research has identified three reasons for the insufficient secretion of C1-INH. Firstly, the coexistence of normal and mutant C1-INH proteins leads to inadequate secretion of C1-INH [12]. Secondly, the secretion of mutant C1-INH is accompanied by an additional oligosaccharide [13] disrupting its original function. Alternatively, certain mutants induce C1-INH polymerization in vitro, rendering it incapable of fulfilling its intended role [14]. The gradual decline in C1-INH secretion over a prolonged period, ultimately leads to the manifestation of HAE disease primarily. Therefore, studying the function of SERPING1 gene, particularly the C1-INH protein, may also be one of the best entry points for exploring the occurrence of HAE.

In this study, a new *SERPING1* gene mutation was found in the previously collected HAE family, and the accumulation of C1-INH protein in cells caused by the mutation and its effect on cell homeostasis was clarified by cell experiments, which is intended to find a new marker for early diagnosis and treatment of HAE.

## Materials and methods

### Patient information collection

The study population consisted of a pedigree affected by HAE, which was recruited from Chengdu City, Sichuan Province. This pedigree included a total of 13 family members. The proband and some of her families, who were diagnosed with HAE in 2016, received the diagnosis at both the Peking Union Medical College Hospital and Sichuan Provincial People’s Hospital. Consequently, all family members underwent measurement of C1-INH and complemented C4 as diagnostic tools for HAE and pathological myopia. The study adhered to the principles outlined in the Declaration of Helsinki and obtained approval from the ethics committee of Sichuan Provincial People’s Hospital (2021. No.465). Additionally, all participants provided informed consent specific to the study.

### Extraction of whole genome DNA

Obtain 5 mL of peripheral blood samples with EDTA anticoagulant, followed by centrifugation to separate the white cell layer. Transfer the white cell layer to a centrifuge tube containing red blood cell lysate, and subject it to thorough agitation. Centrifuge the mixture again to obtain a purified white cell mass. Subsequently, introduce the leukocyte lytic solution and collect the resulting supernatant after vigorous vortexing and centrifugation. Sequentially add isopropanol and anhydrous ethanol, followed by centrifugation to collect and precipitate the desired components. Finally, dissolve the DNA by adding 200 µL of ultra-pure water.

### Targeted capture high-throughput sequencing

The exon region of the entire genome was captured using the sequence capture technique. Initially, the full exon group liquid phase capture technique was employed to efficiently and specifically enrich human DNA in the full exon region. Subsequently, high throughput and high-depth sequencing were performed using the HISEQX10 platform.

### Pathogenic gene screening

The obtained sequencing data were analyzed for information based on the UCSC reference genome (Hg19, 2009), specifically focusing on single sample full exon analysis. The primary analysis types encompassed the evaluation of sequencing data quality, detection of variations, and screening for mutations. Mutations were screened according to the GnomAD database (https://gnomadbroadinstitute.org/), the Thousand Genome Database (NCBI: https://www.internationalgenome.org/), and the internal database composed of normal people. Through literature review and data analysis, the harmful effects of gene mutations on protein structure and function were predicted by the software Polyphen2 (http://genetics.bwh.harvard.edu/pph2/) and Mutation Taster (http://mutationtaster.org/). The protein structure was simulated and predicted by SWISS-MODEL (https://swissmodel.expasy.org/) online protein model prediction software, and the protein structure change and the interaction between amino acids were analyzed by PyMOL software.

### Mutation detection and pedigree analysis

Following the examination of numerous mutations in the database, Sanger sequencing was employed to authenticate the potential pathogenic mutations and isolate mutations identified in each sample from the family, with a particular focus on the *SERPING1* gene mutation. Primers were devised using Primer 3 (http://bioinfo.ut.ee/primer3) based on the human genome database, and all primers employed in this study were synthesized by Sangon Biotech (Shanghai) Co., Ltd. The *SERPING1* gene primers designed by Primer3 (refer to Table 1) were utilized to amplify the specific fragments of the* SERPING1* gene and subsequently purify the PCR products. Subsequently, Sanger sequencing was conducted.

**Table 1:** Sequencing primers for *SERPING1* mutation sites

PRIMER	Sequence (5’ to 3’)
SERPING1-c.708T > G-F	CCCCACAGACAGACAACAT
SERPING1-c.708T > G-R	CCTTTCTCCACAACCTCACC

### Construction of wild-type and mutant plasmids of SERPING1

The pcDNA3.1 empty vector, pcDNA3.1-FLAG-SERPING1-EGFP (NM_001032295) and pcDNA3.1-HA-SERPING1 (NM_001032295) plasmids were generated by the Youbao organism of Hunan Keai Medical Device Co., Ltd., resulting in the production of the wild-type fusion protein of the *SERPING1* gene with FLAG and HA tag at the N-terminal. The pcDNA3.1-FLAG-SERPING1-EGFP and pcDNA3.1-HA-SERPING1 plasmid underwent mutagenesis using the Q5 ^®^site-directed mutation kit (New England Biolabs, USA), resulting in the construction of different point mutation plasmids of the *SERPING1* gene: c.G550A, c.C566A, c.T708G (refer to Table 2). Subsequently, the plasmid was introduced into competent Escherichia coli DH5α (TransGen Biotech biology Corp, China) through instantaneous transformation, followed by extraction of the point mutant plasmid using plasmid extraction techniques. Then the mutated plasmid was sequenced by ABI-3130 sequencer to verify whether the mutation was successful or not.

**Table 2:** Point mutation primers of the *SERPING1* gene

PRIMER	Sequence (5’ to 3’)
c.G550A-F	GGTCCTGCTCAGGGCTGGGGA
c.G550A-R	TGGGTAAGGAGGCTGGCG
c.C566A-F	GGGGAGAACAACAAAACAAACCTGGAG
c.C566A-R	AGCCCCGAGCAGGACCTG
c.T708G-F	GGGACACCTTGGTGAATGCCTC
c.T708G-R	TTATGGCCAGGTCTGGGC

### Cell culture

The 293T cells (human embryonic kidney cells transfected with adenovirus *E1A* gene) utilized in this study were obtained from the Molecular Genetics Center Laboratory of Sichuan People’s Hospital, which is affiliated with the University of Electronic Science and Technology. These cells were cultured in DEME high glucose medium (HyClone, USA) supplemented with 10% fetal bovine serum (Gibco, USA), and maintained in a cell incubator at a temperature of 37 ℃ with a 5% CO_2_ concentration.

### Plasmid transfection

Once the cell density reached approximately 60–70%, the culture medium was aspirated and replaced with an equal volume of serum-free medium. Following adaptation to the serum-free conditions, cell transfection was performed using Opti-MEM (Gibco, USA), Lipofectamine 3000 reagent (Thermo, USA) and RNATransMate (Sangon Biotech (Shanghai) Co., Ltd.). The pcDNA3.1-FLAG-EGFP and pcDNA3.1-HA vector without any content, plasmids expressing the normal *SERPING1* gene, the mutated-*SERPING1* gene and siRNA-GRP75(Sangon Biotech (Shanghai) Co., Ltd.) were individually or together introduced into 293T cells. Subsequently, the cells were incubated at a temperature of 37 ℃ and a concentration of 5% CO_2_ for a duration of 48 h. Once the cell density reached a moderate level, the cells were taken out of the incubator for further experiments. The sequences of the siRNA-GRP75 are shown in Table 3.

**Table 3:** The sequences of the siRNA-GRP75

Primers	Sequence (5’ to 3’)
GRP75#1-F	GCGAUAUGAUGAUCCUGAATT
GRP75#1-R	UUCAGGAUCAUCAUAUCGCTT
GRP75#2-F	AGACAAAUCAGAAGACAAATT
GRP75#2-R	UUUGUCUUCUGAUUUGUCUTT
GRP75#3-F	CCAAGAUGGAAGAAUUCAATT
GRP75#3-R	UUGAAUUCUUCCAUCUUGGTT

### Cell protein collection, concentration determination, and western blot

To ensure the collection of both intracellular and extracellular proteins, 10 cm dishes were utilized for cell culture. To collect extracellular proteins, the supernatant of the cell culture was absorbed, followed by the addition of a protease inhibitor Cocktail. The mixture was then centrifuged at a low speed, and the upper medium was transferred to a MAX cone bottom quick sealing tube. The tube was sealed using a plastic heater, and high-speed centrifugation was performed to collect the precipitation at the bottom of the tube. To collect cellular proteins, RIPA cell lysate (Solarbio, China) containing the Cocktail was added to the cells. The liquid transfer gun was used to repeatedly blow until the cells detached. Ultrasound was then used to break the cells, followed by the addition of 1% SDS. The denatured proteins were heated in a metal bath at 95 ℃. The intracellular and extracellular protein concentrations were determined using the BCA kit (Beyotime, China). The protein samples were subjected to SDS-PAGE electrophoresis and subsequently transferred onto a 0.45 μm PVDF membrane (Solarbio, China). Following sealing with 5% bovine serum albumin for 1.5 h, the membrane was incubated overnight at 4 ℃ with the 1:1000 diluted primary antibody (Proteintech, USA). Subsequently, the TBST membrane was washed three times and incubated with the secondary antibody (Thermo, USA) at room temperature for 1 h. Finally, the protein band was visualized using the ECL luminescent reagent. The cytoplasmic protein levels were normalized to β-ACTIN or GAPDH.

### Immunofluorescence

293T cells were cultured on a 6-well plate. Once the cells reached a specific density, they were fixed using 4% paraformaldehyde, permeabilized with 0.2% Triton, and blocked with 5% bovine serum albumin. Subsequently, the cells were incubated at 4 ℃ with 1:100 diluted primary antibody. Following this, the cells were incubated with secondary antibodies conjugated to Alexa Fluor^®^ 594 or 647 for 1 h (Thermo, USA), and the nucleus was stained with DAPI. Finally, the cells were observed using a confocal microscope. After cells were fluorescence labelled using the fluorescent probe in the JC-1 detection kit (Keygen Biotech, China), confocal microscopy was employed for visualisation and observation. The green dyes indicate the presence of JC-1 monomers within the cytoplasm, and the red dyes indicate the presence of JC-1 aggregates within the nucleus.

### Flow cytometry analysis

We used the Annexin V-FITC/PI apoptosis detection kit (Keygen Biotech, China) to observe the apoptosis of cells that digested with trypsin without EDTA. The cell suspension was washed twice with PBS and centrifuged at 800 rpm for 3 min. The cells were resuspended in 500 µL of binding buffer, Annexin V was added by blowing gently, and propidium iodide was added by mixing gently. Flow cytometry (Beckman Coulter, USA) was used to detect apoptosis after 10 min at room temperature. The enhanced mitochondrial membrane potential assay kit with JC-1 (Beyotime, China) was used to rapidly and precisely detect alterations in the mitochondrial membrane potential (MMP) of cells and the onset of early cell apoptosis. Similar to the above operation, cells were collected and resuspended using a centrifuge, followed by the assessment of JC-1 monomer and JC-1 polymer fluorescence within the cells using flow cytometer (Beckman Coulter, USA).

### ATP detection assay

The Enhanced ATP Assay Kit (Beyotime, China) is used by us to detect intracellular ATP levels. Following the prescribed protocol, 200 µL of cracking solution were added to each well of the 6-well plate. The supernatant was obtained by centrifugation at 4 ºC and 12,000 g for 5 min, and subsequent measurements were conducted. Dilute the ATP standard solution with ATP detection lysis solution to an appropriate concentration gradient and use a chemiluminescence instrument to test and calculate the standard curve. In advance, 100µL of ATP detection working solution should be placed in the detection well and let sit at room temperature for 3–5 min to consume all the background ATP. Subsequently, 20µL of prepared cell lysates were added to the detection well and tested using a chemiluminescence instrument.

### Statistical analysis

All experiments were repeated at least three times to ensure reproducible outcomes. SPSS17.0 software and GraphPad Prism 8.0 package.were used to analyze the data. Exprimental data are expressed as the mean ± standard error of the mean (SEM). The data sets were analyzed using a Shapiro-Wilk test to assess normality. If the data did not conform to a normal distribution, nonparametric tests were used. One-way ANOVA and T-test were used to compare the data among groups. The *P* < 0.05 indicates that there is a significant difference in data between groups, which is expressed by “*”.

## Results

### Clinical and genetic information of the pedigree

**Fig. 1:**
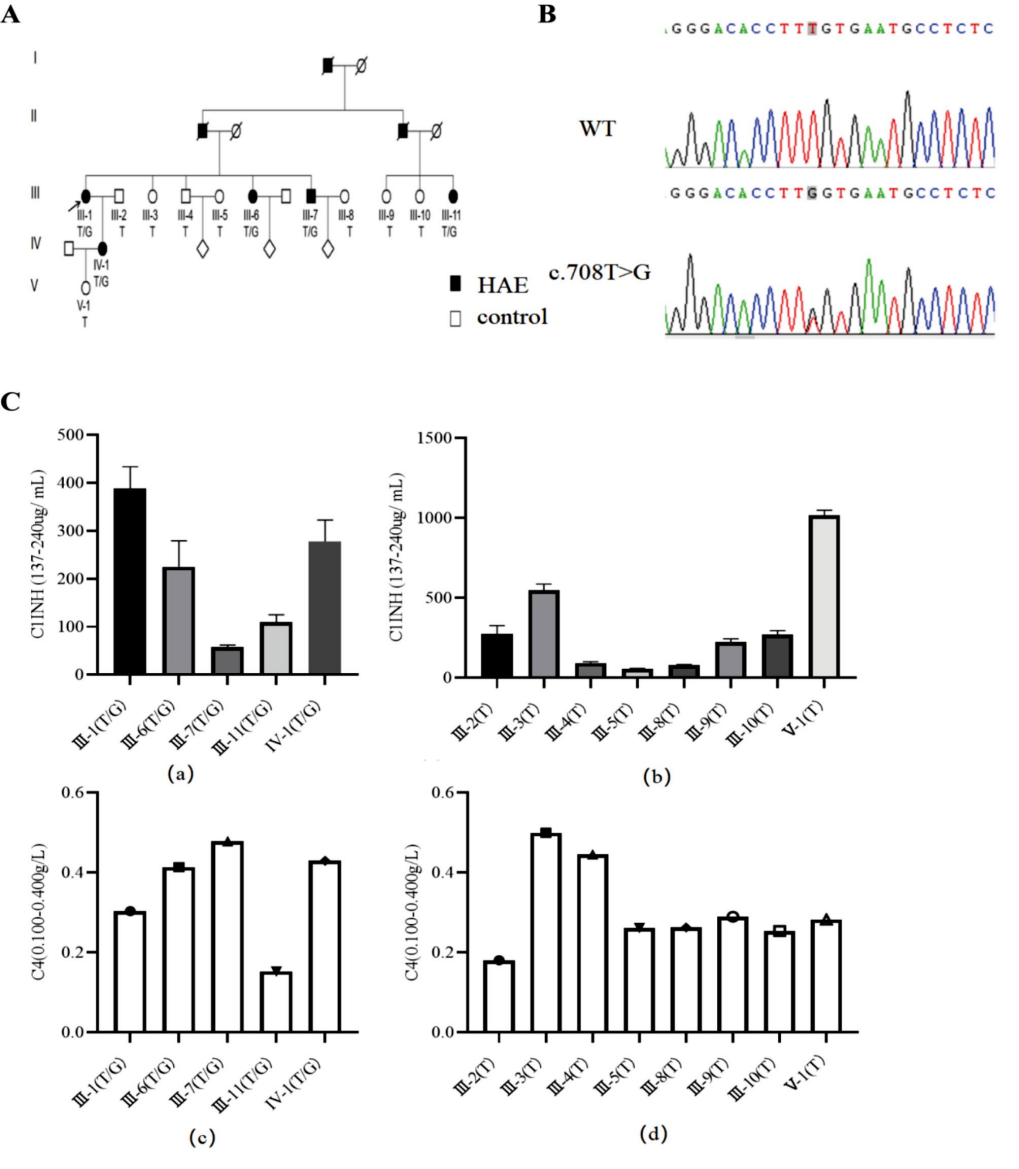
The genetic and clinical information of a family with HAE. (**A**) HAE pedigree map: the black arrow points to the proband, the circle represents the female, the square represents the male, the blackened represents suffering from HAE, and there is a diagonal line on the figure indicating died. (**B**) Sanger sequencing results for the *SERPING1* c.708T > G mutation; (**C**) Concentrations of C1-INH and C4 in peripheral blood of HAE pedigree. a: C1-INH plasma level of HAE patients in the pedigree by ELISA; b: C1-INH plasma level of normal subjects in the pedigree by ELISA; c: C4 plasma level of HAE patients in the pedigree; d: C4 plasma level of unaffected subjects in the pedigree

Thirteen participants were included in the hereditary angioedema pedigree, with five individuals diagnosed with HAE by professional doctors from two authoritative hospitals, including proband III-1 (71 years old, pathological myopia), her daughter IV-1 (32 years old, high myopia), proband’s sister III-6 (60 years old, bronchitis), proband’s younger brother III-7 (63 years old, bronchitis), and proband’s cousin III-11 (64 years old, pulmonary heart abscess) (Fig. 1A). Some normal individuals in this family, such as III-3 (69 years old, bronchitis) and III-4 (51 years old, sleep disorders) were also included in this study. A previously unidentified causative mutation site, c.708T > G (p. Phe236Leu), was detected (Fig. 1B). Heterozygous mutations c.708T > G were found in all five affected members of the pedigree. The presence of this mutation in the family demonstrates co-segregation of genotype and phenotype, indicating an autosomal dominant mode of inheritance. This indicates that patients diagnosed with HAE (III-1, III-6, III-7, III-11, and IV-1) demonstrate uniformity with the same mutation (c.708T > G, p. Phe236Leu).

Their peripheral blood was collected when all patients in the entire family had not developed symptoms, and the levels of C1-INH and C4 in plasma were detected. We found that patients III-7 and III-11 exhibited lower levels of C1-INH compared to the established normal range (137–240 µg/mL). Conversely, patients with III-1 and IV-1 displayed higher levels of C1-INH than the normal range. The majority of unaffected individuals in the pedigree demonstrated C1-INH levels within the normal range, except III-5, whose levels were lower than normal, and III-3 and V-1, whose levels were higher than normal. Regarding C4 levels, III-1 and III-11 fell within the normal range (0.100–0.400 g/L), while III-6, III-7, and IV-1 exhibited levels higher than the normal range. Most of the unaffected subjects had normal C4 levels, except that III-3 and III-4 were above the normal range (Fig. 1C). There have been many previous literature reports that the expression of complement in HAE patients may be related to various factors such as the onset time, site of onset, patient status, medication history, and whether the disease has occurred [15–19]. The HAE patients from the same family we collected did not show any symptoms at the time of sample collection, and there were also many differences among them (lifestyle, disease status, medication history, etc.). Therefore, as our research results show, there was no consistency in the levels of C1-INH and C4 among patients with the same mutation in the same family we collected. This result can also indicate that it is difficult to accurately diagnose HAE based solely on the levels of C1 INH and C4.

### C1-INH (Encoded by *SERPING1* gene) amino acid mutation sites conservation analysis

**Fig. 2:**
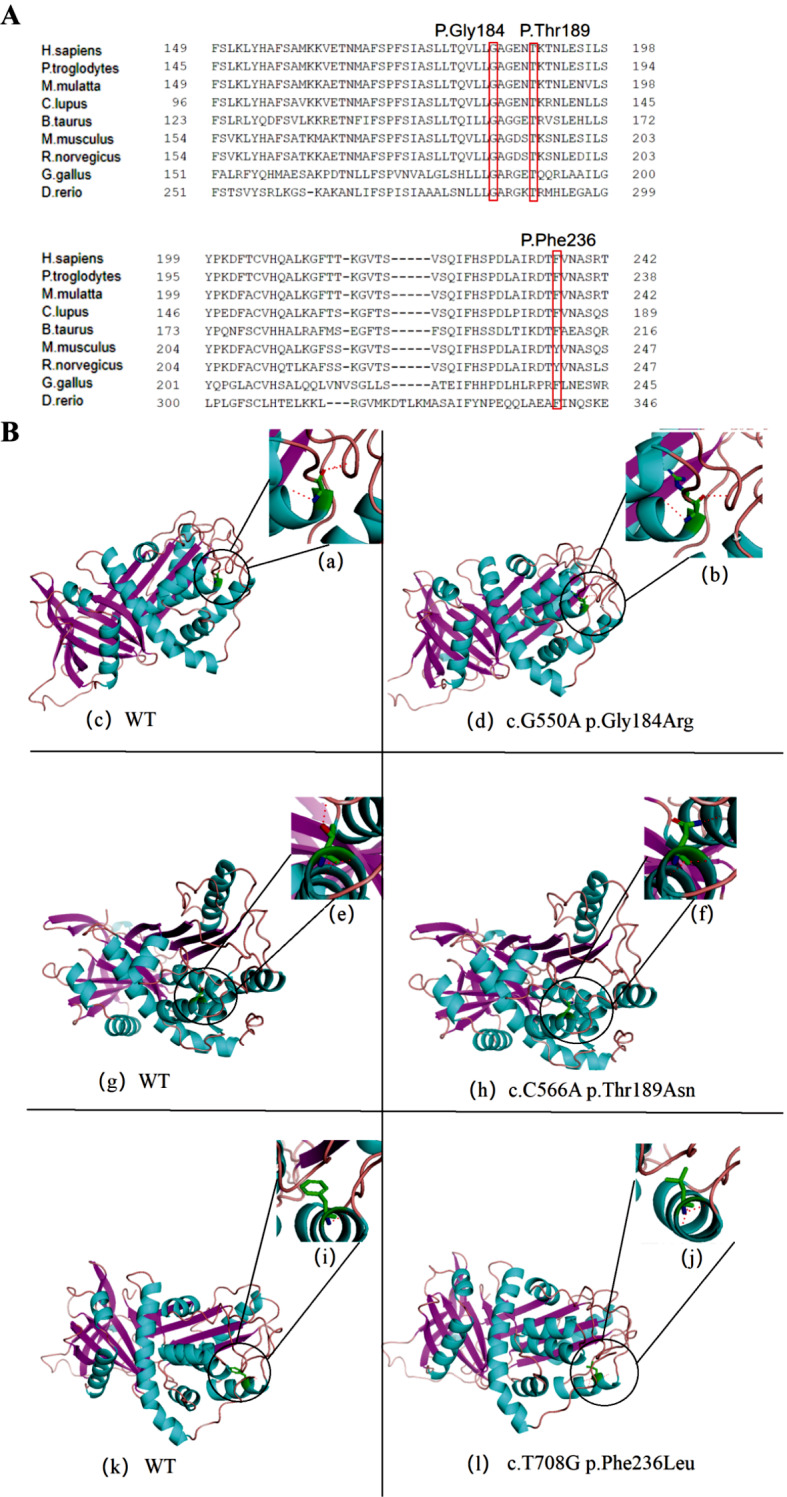
C1-INH protein amino acid mutation sites conservation analysis. (**A**) Conservation analysis of protein amino acid mutation site of *SERPING1* gene. (**B**) Structural models of wild type and mutant proteins of *SERPING1* gene: Blue represents the alpha helix of protein structure, purple represents the β-fold of protein structure, and pink represents the ring of protein structure. The green “stick model” is the molecular structure model of the amino acid residues of the studied site, and the red “stick model” is the molecular structure model of the hydrogen bond which may be combined by the amino acid residues of the studied site

The c.708T > G mutation site, along with the c.550G > A and c.566C > A mutation site of HAE caused by *SERPING1* gene mutation reported in previous literature, was chosen for analysis of the protein’s structure and function. Through comparison of amino acid sequences across various species, it was observed that all three mutation sites were located in highly conserved positions within the protein (Fig. 2A). Furthermore, the utilization of PolyPhen2 and MutationTaster software predicted that these three mutation sites within the *SEERPING1* gene would have detrimental impacts on the protein’s functionality (Table 4).

**Table 4:** Preliminary analysis of mutation sites of *SERPING1* gene

Base mutation	Amino acid change	PolyPhen-2^a^	Mutation Taster^b^
c.550G > A	Gly184Arg	1.00 Probably Damaging	0.999 disease-causing
c.566C > A	Thr189Asn	/	1.000 disease-causing
c.708T > G	Phe236Leu	0.999 Probably Damaging	0.899 disease-causing

*Note*^a^ In PolyPhen-2 prediction, the closer the score is to 1, the greater the harmfulness probability of mutation is; the closer the score is to 0, the smaller the harmfulness probability of mutation is. ^b^ In Mutation Taster prediction, the closer the value is to 1, the higher the prediction accuracy is, and “disease causing” means it may be harmful

Furthermore, the protein structures of both wild-type and mutants were predicted using SWISS-MODEL and Pymol software. The findings indicated that these missense mutations did not have a substantial impact on the overall spatial structure of the protein. However, the c.550G > A mutation resulted in the substitution of the 184th polar uncharged glycine with the polar positively charged arginine, thereby inducing a modification in the amino acid’s structure. Similarly, the c.566C > A mutation led to the replacement of the 189th polar uncharged threonine with the polar uncharged aspartic acid, resulting in a structural alteration of the amino acid. Additionally, the c.708T > G mutation caused the nonpolar hydrophobic amino acid phenylalanine at position 236 to be substituted with nonpolar leucine, consequently disrupting the hydrogen bond that should normally connect with other amino acids, thus affecting the function of proteins (Fig. 2B).

### Effect of *SERPING1* mutations on the expression of extracellular and extracellular C1-INH

**Fig. 3:**
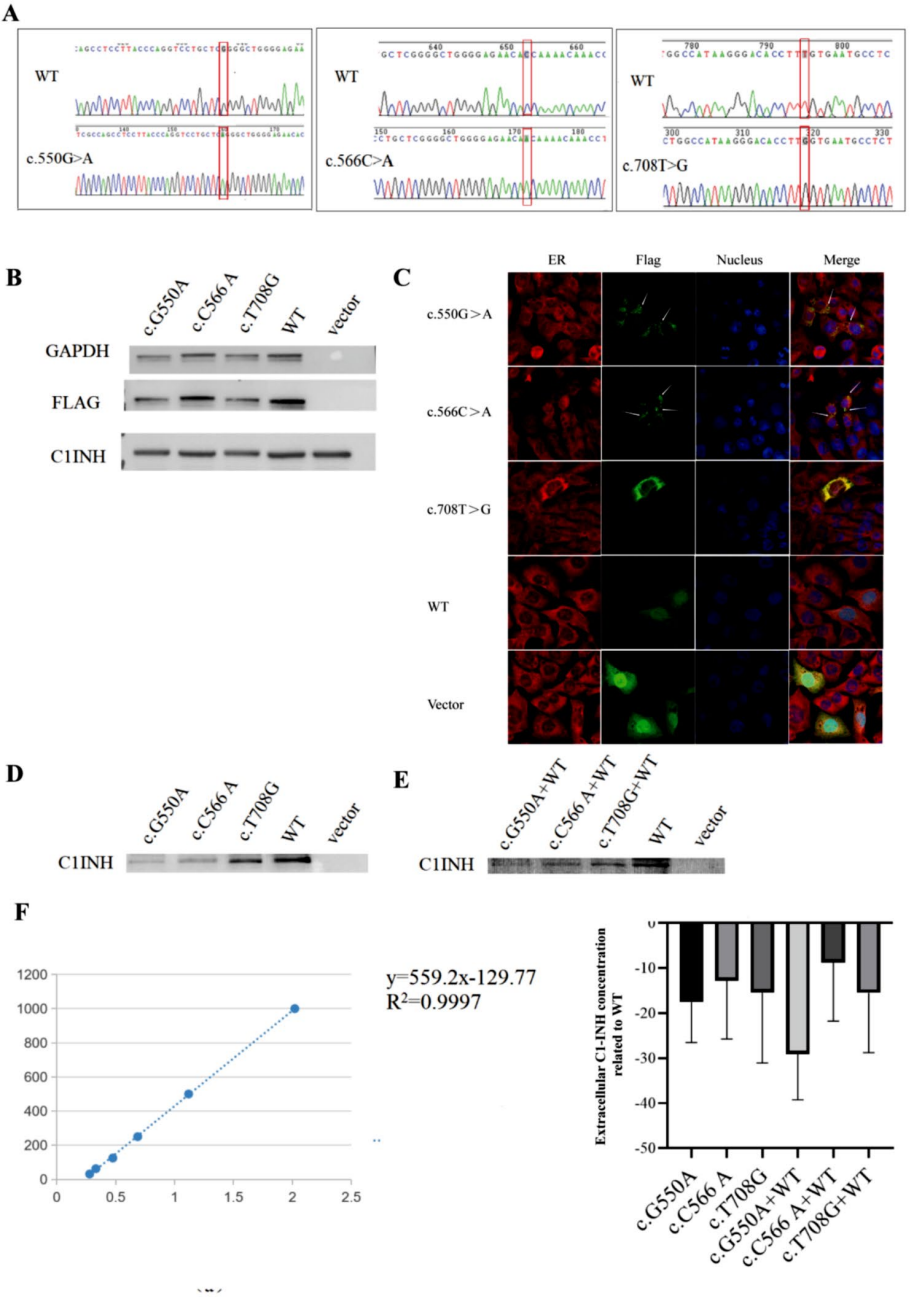
Effect of *SERPING1* mutations on the expression of extracellular and extracellular C1-INH. (**A**) *SERPING1* gene mutation plasmid sequencing: The red box shows c.550G > A, c.566C > A and c.708T > G mutant bases. (**B**) Detection of FLAG and C1-INH protein expression after plasmid transfection by Western blot. (**C**) Protein subcellular localization of WT and mutant of *SERPING1* gene: Red indicates the location of the endoplasmic reticulum, green indicates the location of the C1-INH protein through the FLAG tag, blue indicates the nucleus, and the white arrow indicates the accumulation of proteins in the cell. (**D**-**E**) Western blot detection of protein expression of extracellular C1-INH after single transfection of mutant plasmid or co-transfection of mutant plasmid + WT. (**F**) Left: C1-INH protein concentration standard curve; Right: The difference of C1-INH protein concentration between mutant and wild type detected by ELISA. These experiments in **B**-**F** were replicated 3 times with similar results

The wild-type plasmids (WT) and mutant plasmids of pcDNA3.1-FLAG-SERPING1-EGFP (Fig. 3A) were successfully transfected into 293T cells with good growth. In contrast, 293T cells transfected with an empty vector did not express C1-INH protein, indicating the absence of endogenous C1-INH production. However, C1-INH protein was detected in 293T cells transfected with both WT and mutant plasmids, confirming the successful transfection of the plasmid (Fig. 3B).

C1-INH is secreted via the endoplasmic reticulum (ER), and the *SERPING1-*mutation-induced structural alteration of C1-INH facilitates its binding to the target protease, leading to the accumulation of polymers in ER and consequent reduction in secretion. Hence, immunofluorescence was employed to examine the localization of C1-INH in ER. The results revealed a significant aggregation of the protein in ER transfected with the mutant plasmid, particularly c.708T > G mutation when compared to WT (Fig. 3C). Additionally, the protein level of extracellular C1-INH was determined in this study. Western blot analysis revealed a significant decrease in the expression of extracellular C1-INH protein in samples with c.550G > A, c.566C > A, c.708T > G mutations alone (Fig. 3D), as well as in samples with co-transfection of c.550G > A + WT, c.566C > A + WT, and c.708T > G + WT (Fig. 3E), compared to the WT group. These findings were further supported by the results obtained from ELISA (Fig. 3F). Therefore, it can be inferred that c.708T > G mutations, similar to c.G550A and c.C566A mutations, may result in the aggregation of C1-INH in the endoplasmic reticulum, leading to impaired secretion.

### *SERPING1* mutations upregulated GRP75, resulting in cellular calcium overload and mitochondrial damage

**Fig. 4:**
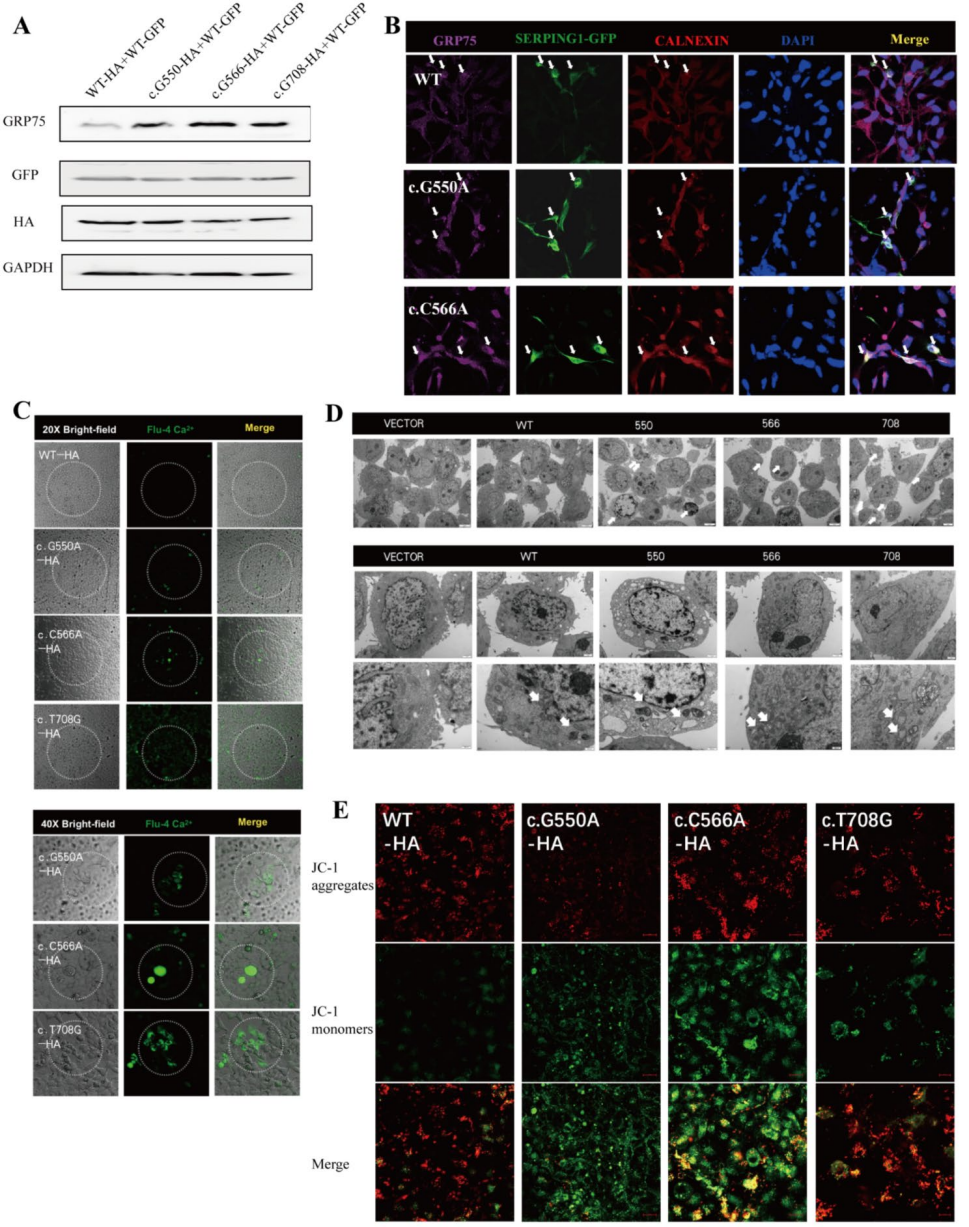
*SERPING1* mutations upregulated GRP75, resulting in cellular calcium overload and mitochondrial damage. (**A**) The GRP75 expression by co-transfecting with WT-HA + WT-GFP, c.G550A-HA + WT-GFP, c.C566A-HA + WT-GFP, and c.T708G-HA + WT-GFP. (**B**) Co-localization of GRP75 and SERPING-1 in endoplasmic reticulum detected by immunofluorescence: Purple indicates GRP75, green indicates SERPING-GFP fluorescent protein, red indicates endoplasmic reticulum, blue indicates nucleus, and white arrow points to co-location. (**C**) The level of intracellular Ca^2+^ was observed under 20X and 40X visual field after single transfection of c.G550A, c.C566A, c.T708G and WT plasmid. (**D**) Transmission electron microscope observation of mitochondrial morphology of cells after single transfection of c.G550A, c.C566A, c.T708G and WT plasmid. (**E**) JC-1 detection of mitochondrial membrane potential after single transfection of c.G550A, c.C566A, c.T708G and WT plasmid. These experiments in **A**-**E** were replicated 3 times with similar results

Previous research has demonstrated that the presence of Ca^2+^ leads to an increased affinity between C1-INH and GRP75, a crucial protein located at the junction of the endoplasmic reticulum and mitochondria. This protein facilitates the transportation of Ca^2+^ from the endoplasmic reticulum to the mitochondria [20–23]. Through Western blot analysis, we have confirmed that the co-transfection of mutant c.550G > A + WT, c.566C > A + WT, or c.708T > G + WT significantly enhances the expression of GRP75 (Fig. 4A). Additionally, immunofluorescence analysis revealed that the transfection of mutant c.550G > A, c.566C > A, and c.708T > G also up-regulated the expression of GRP75 and promoted the colocalization of GRP75 and C1-INH on the endoplasmic reticulum (Fig. 4B). The findings indicated that the presence of C1-INH in the endoplasmic reticulum may enhance the expression and binding of GRP75 protein. To further investigate this, the concentration of Ca^2+^ within the cells was examined using fluorescence microscopy. It was observed that the *SERPING1* mutation led to an increase in intracellular Ca^2+^ concentration, particularly following transfection of c.566C > A and c.708T > G (Fig. 4C). Cellular Ca^2+^ overload frequently results in the impairment of mitochondrial structure and function. Indeed, electron microscopy revealed mitochondrial atrophy in cells transfected with the mutant plasmid (Fig. 4D). JC-1 detection demonstrated that transfection of the three mutant plasmids caused a decrease in mitochondrial membrane potential (Fig. 4E). These findings suggested that the c.708T > G mutation shares similarities with the c.550G > A and c.566C > A mutations, which induce up-regulation of GRP75, resulting in Ca^2+^ overload and subsequent mitochondrial damage.

**Fig. 5:**
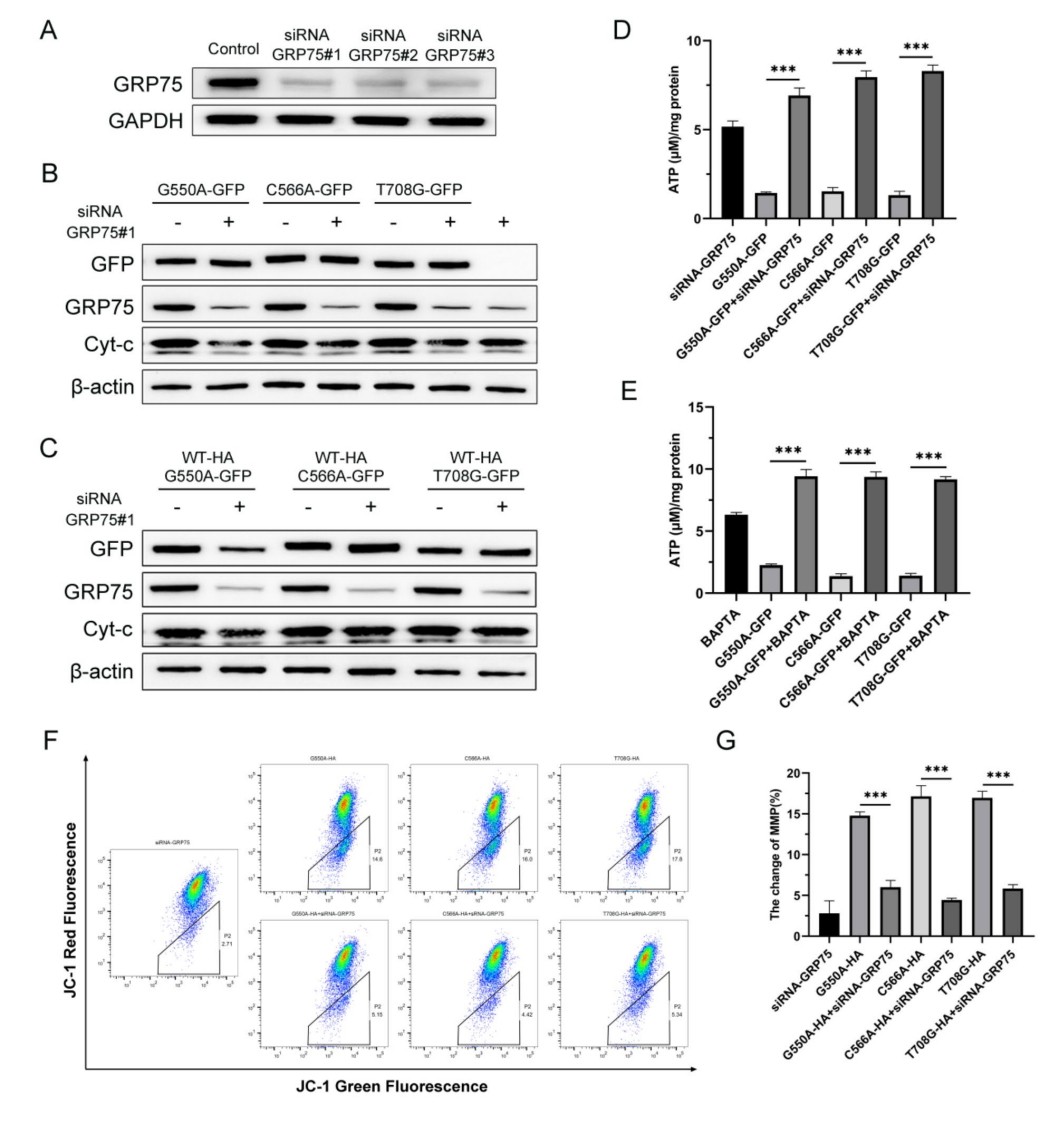
Down-regulating GRP75, rescuing in cellular calcium overload and mitochondrial damage. (**A**) Validation of the efficiency of knockout for siRNA targeting GRP75. (**B**-**C**) Decreased expression of GRP75 contributes to a reduction in the release of Cytochrome C (Cyt-C). (**D**) Reduced GRP75 expression help to improve cell ATP synthesis recovery. (**F**-**G**) Flow cytometry was used to observe the changes in mitochondrial membrane potential (MMP). When down-regulate, the expression of GRP75 ameliorated changes in MMP. These experiments in **A**-**E** were replicated 3 times with similar results. Representative data in **A**-**G** are presented as mean ± SEM; ***, *P* < 0.001

According to the findings presented above, it can be inferred that the GRP75 protein plays a role in facilitating the retention of C1-INH protein within the endoplasmic reticulum. In order to further explore the specific role of GRP75 protein in this process, we then used siRNA technology to knock down endogenous GRP75 expression in cells. Three siRNA fragments were constructed, and siRNA-GRP75 #1 was chosen for further experiments based on its confirmed knockdown efficiency using Western blot analysis (Fig. 5A). Based on prior experimental evidence, it has been established that mutations in C1-INH can elicit cellular state abnormalities, including alterations in mitochondrial membrane potential, calcium ion levels, and cellular microstructure. Monitoring cytochrome C protein (Cyt-C) secretion, quantifying cellular ATP levels, and assessing membrane potential with JC-1 are essential diagnostic markers frequently employed in characterising the aforementioned cellular state abnormalities. Consequently, we opted to co-transfect the mutant plasmid with siRNA-GRP75 in order to investigate potential alterations in cellular phenotype. The experimental findings indicated that co-transfection of the mutant plasmid with siRNA-GRP75 into cells resulted in a significant improvement in cell state, characterized by decreased Cyt-C release (Fig. 5B-C). Moreover, decreased expression of GRP75 could help the cells, which over-expressed the C1-INH mutant, enhance ATP synthesis recovery. Reducing the expression of GRP75 promotes the recovery of cellular ATP synthesis, similar to using calcium chelating agents (BAPTA, calcium scavengers) (Fig. 5D-E). Additionally, the study revealed that down-regulating GRP75 ameliorated changes in MMP and reduced cellular apoptosis (Fig. 5F).

### *SERPING1* mutations induced cell apoptosis

**Fig. 6:**
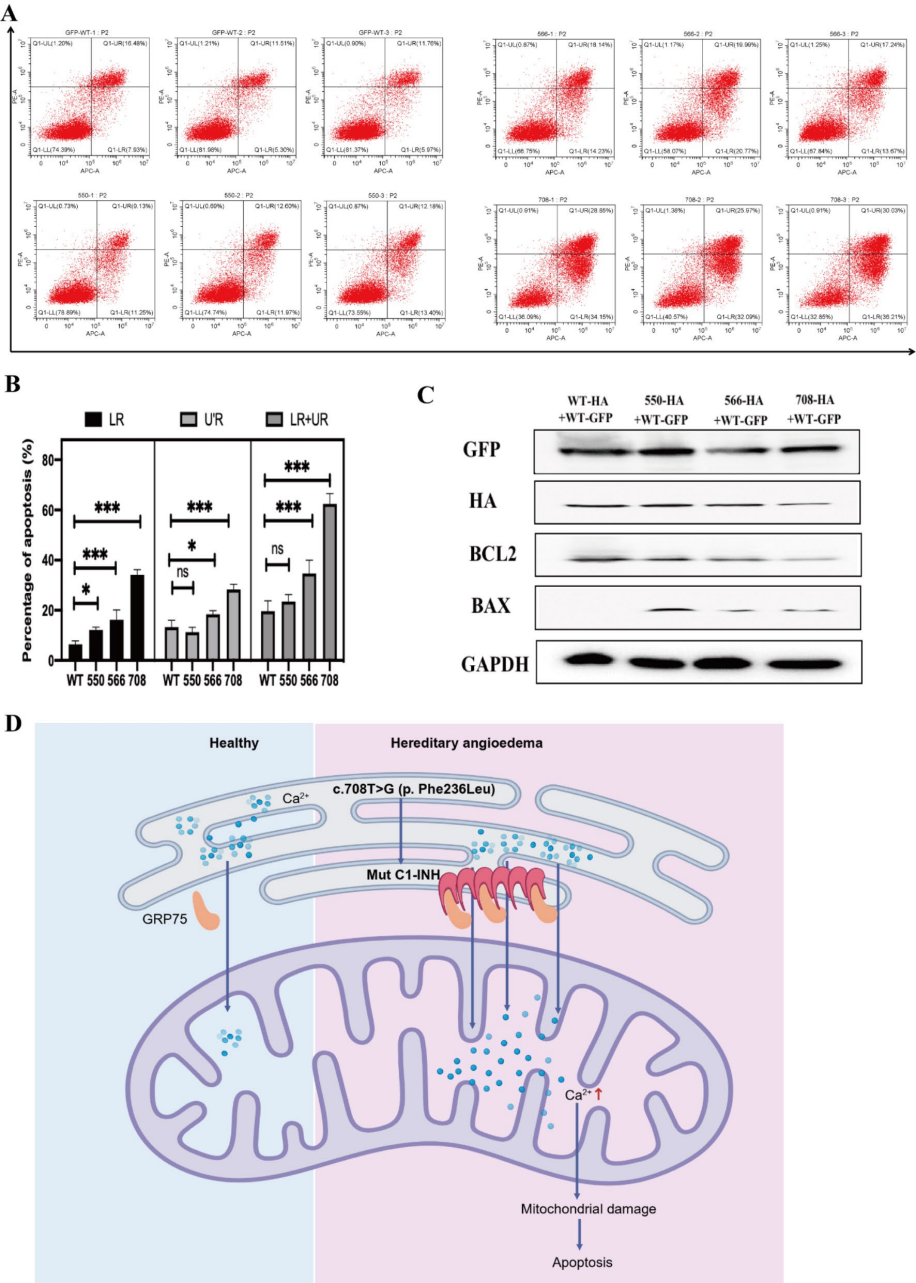
*SERPING1* mutations induced cell apoptosis. (**A**) Flow cytometry was used to observe the apoptosis after single transfection of c.G550A, c.C566A, c.T708G and WT plasmid. (**B**) Statistical results of cell apoptosis by flow cytometry. (**C**) The BAL2 and BAX expression after co-transfecting with WT-HA + WT-GFP, c.G550A-HA + WT-GFP, c.C566A-HA + WT-GFP, and c.T708G-HA + WT-GFP. (**D**) Schematic diagram of apoptosis caused by c.708T> G mutation in hereditary angioedema. All experiments were independently performed at least three times to ensure repeatable results. Representative data in E and F are presented as mean ± SEM; *, *P* < 0.05; **, *P* < 0.001; ***, *P* < 0.001

Mitochondrial damage plays a crucial role in the induction of apoptosis. To investigate this phenomenon, we employed Annexin V and PI as markers to identify apoptotic cells and quantified the percentage of apoptosis using flow cytometry. As anticipated, the co-transfection of three mutant plasmids along with the WT plasmid resulted in a substantial increase in apoptosis, with the c.708T > G + WT mutant plasmid combination exhibiting the most pronounced effect (Fig. 6A-B). Furthermore, Western blot analysis revealed an up-regulation of the pro-apoptotic protein BAX and a down-regulation of the anti-apoptotic protein BCL2 following transfection with mutant plasmids (Fig. 6C). The above results suggest that *SERPING1* mutations lead to significant apoptosis.

## Discussion

HAE is a rare hereditary disease primarily attributed to abnormal C1-INH concentration or function resulting from genetic variation in the *SERPING1* gene. To date, over 500 variants of the *SERPING1* gene associated with HAE have been identified, although their underlying mechanisms remain unclear. This study presented the discovery of a novel HAE pathogenic mutation in clinically diagnosed HAE patients, c.708T > G (p. Phe236Leu), which has not been previously reported. Additionally, the study revealed that the c.708T > G mutation shares similarities with c.550G > A and c.566C > A mutations, leading to the accumulation of mutant C1-INH in the endoplasmic reticulum, activation of GRP75, and mediation of calcium overload, mitochondrial damage, and eventually apoptosis (Fig. 6D, draw with Biorender, agreement number: JC262J2A8I).

In this study, five patients with HAE exhibited typical clinical manifestations and family history and were diagnosed strictly following the international WAO/EAAC1 management guidelines. However, in detecting C1-INH and C4 in patients, the plasma C1-INH and C4 levels in some patients (III-1, III-6, and IV-1) were not lower than those of normal subjects. The analysis of the reasons may be due to the particularity and unpredictability of HAE. As mentioned before, we collected blood samples from HAE families at the same time but not during the acute onset period, and there were significant differences among patients and normal in this HAE family (gender, living area, lifestyle habits, age of onset, and history of medication). The levels of C1-INH and/or C4 are also important reactants in the acute phase, and in the case of infection or inflammation, both the levels of C1-INH and C4 could be altered a lot. When some patients have a history of chronic diseases, colds, and other symptoms, the secretion of immune factors or C1-INH and C4 in the patient’s body may be affected by drugs and inflammatory signals, leading to corresponding changes [24]. In this HAE family, patients have an age span of over 30 years and with entirely different long-term chronic inflammation-related diseases (bronchitis pathological myopia, etc.). The proband in this study was previously diagnosed with HAE several years ago and had a prolonged record of using multiple medications to avoid HAE (Danazol). At the same time, patients (III-1, III-6 and III-7, III-11, etc.) and normal (III-3 and III-4) also have different histories of long-term use of different types of drugs [24]. The administration of different drugs can result in alterations in patient detection indicators. Therefore, in the absence of HAE disease, C1-INH and/or C4 expression is exceptionally prone to various situations.

Our results and multiple literature reports mentioned earlier have revealed individual and temporal differences in the levels of C1 INH and C4 in the plasma of different patients under various states [15–19]. Therefore, by solely examining the levels of peripheral blood C1-INH and C4, HAE patients may mistakenly perceive them as “normal”, ultimately leading to a missed diagnosis or delayed diagnosis. Based on our findings and previous literature, we can conclude that the assessment of HAE status based on peripheral blood C1 INH and C4 levels is not entirely reliable. Furthermore, the determination of complement indexes in blood often encounters certain ambiguities. Recent research has indicated that the inclusion of functional diagnosis of complement C1 enhances the reliability of detection [25, 26]. In short, the clinical presentations of HAE are intricate and varied, and the existing diagnostic markers should not be solely relied upon for accurate disease identification, particularly in patients with a complex medication history [27]. Consequently, the exploration of novel diagnostic indicators for HAE is currently a significant area of research.

Recent investigations have revealed that the majority of pathogenic mutations in the *SERPING1* gene typically result in the insolubility of C1-INH proteins within the ER [28, 29]. Similarly, our study also observed that the c.708T > G mutation can induce a substantial accumulation of C1-INH within the ER, resulting in a decrease in C1-INH secretion. Prior research has demonstrated that a reduction in the secretion of C1-INH protein to a particular threshold is associated with a substantial increase in the occurrence of HAE [10, 11]. Notably, this process may coincide with the manifestation of prodromal symptoms. Individuals affected by HAE commonly experience prodromal symptoms such as migraines, fever, cognitive alterations, inattention, memory impairment, and other manifestations [30–32]. Consequently, accurate prediction and timely intervention during this prodromal phase can effectively mitigate the risks associated with HAE. The progressive accumulation of C1-INH in cells can be considered a significant indicator of precursor symptoms for HAE and a crucial approach for preventing and managing the onset and progression of HAE.

Prior research has primarily concentrated on the activation of bradykinin due to inadequate secretion of C1-INH, leading to the development of several efficacious medications for post-HAE attacks [33, 34]. Nonetheless, there is a dearth of studies investigating the prodromal phase of HAE. The period of C1-INH retention is considered to be the initial phase of the disease. To replicate intracellular C1-INH retention in vitro, we utilized the SERPING1-WT/Mut plasmid. The mutant plasmid contained a novel mutation c.708T > G, while two pathogenic *SERPING1* mutations (c.550G > A and c.566C > A) previously documented in the literature were employed as positive controls [35]. Our findings indicate that the mutagenic effect of c.708T > G is comparable to that of c.550G > A, resulting in an elevation of Ca^2+^ levels and mitochondrial damage, ultimately leading to significant apoptosis. Knocking down the expression of RGP75 protein can significantly alter the aforementioned cellular abnormalities. The disruption of cellular homeostasis resulting from protein accumulation in the ER has been established in numerous diseases. This investigation also revealed that an abundance of mutant C1-INH aggregations on the ER induces excessive Ca^2+^ through the GRP75, potentially serving as a noteworthy characteristic during the prodromal phase of HAE.

It is crucial to acknowledge the limitations of this study. Firstly, although blood samples were obtained from the HAE family and C1-INH levels were assessed, there is a dearth of tissue-C1-INH detection, necessitating further refinement in future research. Secondly, we posit that the assessment of intracellular calcium concentration holds potential as a predictive marker for HAE; however, its clinical validation is currently lacking, necessitating future improvements. Moreover, this study fails to investigate the interplay between C1-INH and GRP75, which serves as the intermediary in C1-INH accumulation and mitochondrial impairment. Lastly, given the scarcity of HAE cases, the limited size of the clinical sample incorporated in this study warrants a cautious interpretation of the findings.

In summary, this study provides a comprehensive analysis of the phenomenon wherein the accumulation of C1-INH in cells triggers intracellular alterations before the manifestation of HAE. The findings of this investigation indicate that C1-INH has the potential to modulate and enhance the expression of GRP75, a crucial protein involved in calcium transportation between the ER and mitochondria. Concurrently, the continuous accumulation of C1-INH in the ER leads to disturbances in cellular calcium content, ultimately resulting in the initiation of Ca^2+^-dependent apoptosis.

## Conclusion

This study has successfully identified a novel *SERPING1* pathogenic mutation in HAE, c.708T > G. This mutation has been found to induce the accumulation of C1-INH in the endoplasmic reticulum, subsequently activating GRP75 and resulting in Ca^2+^ overload and apoptosis. These significant findings hold crucial clinical implications for the prognostication and early intervention of HAE.

## Data Availability

The authors confirm that the data supporting the findings of this study are available by contacting the corresponding author. Mutation screening was conducted based on the GnomAD database (https://gnomad.broadinstitute.org/) and the 1000 Genomes Project database (NCBI). Meanwhile, protein structure prediction and simulation were accomplished using tools such as Polyphen2 (http://genetics.bwh.harvard.edu/pph2/), Mutation Taster (http://mutationtaster.org/), and SWISS-MODEL (https://swissmodel.expasy.org/).
