# Correction To: Uncovering a novel SERPING1 pathogenic variant: insights into the aggregation of C1-INH in hereditary angioedema

**DOI:** 10.1186/s13023-025-03572-z

**Published:** 2025-02-06

**Authors:** Lingxi Jiang, Chao Dai, Suyang Duan, Tingting Wang, Chunbao Xie, Luhan Zhang, Zimeng Ye, Xiumei Ma, Yi Shi

**Affiliations:** 1 Sichuan Provincial Key Laboratory for Human Disease Gene Study and the Center for Medical Genetics, Department of Laboratory Medicine, Sichuan Academy of Medical Sciences & Sichuan Provincial People’s, Hospital, University of Electronic Science and Technology of China, 32 The First Ring Road West 2, Chengdu, 610072 Sichuan China; 2 Department of Ophthalmology, Sichuan Provincial People’s Hospital, University of Electronic Science and Technology of China, Chengdu, 610072 China; 3 Research Unit for Blindness Prevention of Chinese Academy of Medical Sciences (2019RU026), Sichuan Academy of Medical Sciences and Sichuan Provincial People’s Hospital, Chengdu, 610072 China; 4 School of Medicine, University of Sydney, Camperdown, 2006 NSW 2050 Australia; 5 Health Management center, Sichuan Provincial people’s Hospital, Chengdu, 610072 China

Following the publication of the recent article in Orphanet J Rare Dis [1] we wish to bring the following corrigendum to your attention. In the above paper, the c.708T > G variant in the SERPING1 gene was initially described as a novel pathogenic variant for hereditary angioedema (HAE). Upon review, we discovered that this variant was actually first reported by Chinese scholars, including Wang Xue, in a Chinese family in 2022 [2]. Currently, the c.708T > G variant is listed in two publicly accessible online databases: ClinVar (VCV001299720.1) and LOVD (#0000878385). Additionally, we utilized the InterVar tool, recommended by the ACMG guidelines, for clinical interpretation of genetic variants, and the SERPING1 c.708T > G variant was classified as Uncertain significance.

Although we were not the first team to report this variant, our study has provided in-depth insights into the underlying mechanism by which the c.708T > G variant mediates the accumulation of C1-INH within the endoplasmic reticulum (ER), subsequently upregulates GRP75 protein expression, and ultimately triggers cellular calcium overload, mitochondrial damage, and apoptosis. Furthermore, we have proposed intracellular calcium concentration as a potential biomarker for predicting acute exacerbations in HAE patients.

We sincerely apologize for not accurately citing the original source of this variant and for failing to conduct an ACMG classification in our paper. However, it is important to note that all research findings and conclusions presented in the paper remain unaffected by this correction, preserving their original scientific value and accuracy.
